# Processed meat products and snacks consumption in ADHD: A case–control study

**DOI:** 10.14744/nci.2021.64497

**Published:** 2022-07-08

**Authors:** Sumeyye Akin, Fatih Gultekin, Ozalp Ekinci, Arzu Kanik, Busra Ustundag, Bilge Didem Tunali, Mohammad B. Abdulrazzaq Al-Bayati, Cemre Yasoz

**Affiliations:** 1 Department of Medical Biochemistry, University of Health Sciences Turkey, Istanbul, Turkiye; 2 Department of Child and Adolescent Psychiatry, University of Health Sciences Turkey, Istanbul Erenkoy Training and Research Hospital for Psychiatry and Neurological Diseases, Istanbul, Turkiye; 3 Department of Biostatistics and Medical Informatics, University of Health Sciences Turkey, Istanbul, Turkiye

**Keywords:** Attention-deficit hyperactivity disorder, children, chocolate, processed meat products, snack, sweets

## Abstract

**OBJECTIVE:**

Attention-deficit hyperactivity disorder (ADHD) has been linked to “unhealthy” food consumption, but the studies in this area are insufficient. The aim of this study is to investigate the relationship between ADHD/related symptoms and processed meat products and snack consumption.

**METHODS:**

This study was conducted on 390 children aged between 6 and 17 with 169 ADHD (38 Girls, 131 Boys) and 221 healthy controls (93 Girls, 128 Boys). Food consumption was evaluated by a modified food frequency questionnaire, including 18 food containing processed meat products and snacks. ADHD symptoms were evaluated by the teacher and parent Turgay DSM-IV-Based Child and Adolescent Disruptive Behavioral Disorders Screening and Rating Scale (T-DSM-IV-Scale) and Conners’ Rating Scale (CPRS, CTRS).

**RESULTS:**

Children with ADHD consumed more processed meat products, milk-based desserts, and chocolate-sweets than controls (p<0.05). A positive correlation was observed between the ADHD symptom scores and the consumption amount of all snacks, the amount of chocolate-sweets consumption, the frequency of consumption of sujuk, chocolate, jelly beans, sweets, cakes, and chocolate spread (p<0.05).

**CONCLUSION:**

Children with ADHD consume more foods rich in saturated fat and sugar than their healthy peers. Processed food consumption of children with ADHD may be associated with ADHD symptoms.

## Highlight key points

- Children with ADHD consume more processed meat products and snacks than healthy peers.
- Children with ADHD are at risk for unhealthy nutrition.
- Consuming processed foods is positively correlated with the presence of ADHD symptoms.

Attention-deficit and hyperactivity disorder (ADHD) is one of the most common psychiatric diseases worldwide, affecting 5–12% of children. ADHD is a neurodevelopmental disorder characterized by inattention, distraction, restless excessive activity, impulsivity, and other deficiencies of executive functions [1].

The role of nutrition in the etiology of ADHD is a topic under investigation. Although many studies have been conducted on nutrients and their components, the results are inconsistent with each other. Common findings in studies investigating the relationship between diet models and ADHD has been associated with unhealthy dietary models (rich in saturated fat-refined sugar and poor in vegetables and fruits) [2]. In a study, those who were newly diagnosed with ADHD were found to consume confectionery, cola drinks, and soft drinks more; fatty fish, fruits, and vegetables less [3]. In a comprehensive prospective study, the data collected about diet models during pregnancy and at certain ages of the child were analyzed, and it was revealed that the unhealthy diet form was associated with an increased risk of internalizing and externalizing behavior [4].

ADHD diagnostic criteria include symptoms such as forgetfulness and losing something easily [1]. Studies are showing that a diet rich in high fat and sugar can have negative effects on memory, learning, and attention [5–11].

Impulsivity symptoms are among the core diagnostic symptoms of ADHD [1]. Western diet has been reported to trigger hyperactivity and impulsivity in adolescent male rats [12]. In a cross-sectional study conducted in adults in the form of an online questionnaire, it was reported that impulsivity was also high in people with fast food consumption [13].

This work was planned as a case–control study to comparing the consumption of processed meat products and snacks with and without ADHD.

## MATERIALS AND METHODS

### Ethics Approval

All procedures contributing to this work comply with the ethical standards of the relevant national and institutional committee on human experimentation with the Helsinki Declaration of 1975, as revised in 2008. The study protocol was approved by the University of Health Sciences Hamidiye Scientific Research Ethics Committee (project/decision no: 19/16).

### Study Design

The present study was a case–control study.

The questionnaires for the case group were conducted by a dietitian face to face. In the control group, the questionnaires were delivered to parents through teachers. The questionnaires from the parents were checked and filled in again by the dietitian with the children. The demographic characteristics of the child and family, frequency, and quantity of consumption of 18 foods, including processed meat products and snacks, were questioned in both groups. The family and the teacher were asked to fill the scales used to evaluate the ADHD findings of the case group, and the forms were taken 1 month later.

### Setting

The recruitment and data collection were ongoing from October 2018 to May 2019. The case group of the study was conducted in the child and adolescent psychiatry clinic in Istanbul. The control group from primary, middle, and high schools in Istanbul were included in the study.

### Participants

The case group was included in 169 ADHD children aged 6–17-year old. The inclusion criteria were as follows (1) normal intelligence based on either a Wechsler Intelligence Scale for Children-full-scale intelligence quotient (WISC-R full-scale IQ) score above 80 or the average/above average academic performance documented with the last year’s final school grades (normal intelligence was confirmed by at least one faculty member of child psychiatry/pediatric neurology), (2) evaluation by a child and adolescent psychiatry clinic for behavioral/learning problems, (3) ADHD diagnosis and subtype according to the DSM-V by a structured psychiatric interview.

In the control group, the questionnaire was conducted on 400 healthy children aged 6–17 years, and 221 people who completed the questionnaires were included in the study. The parents were asked in writing, “Does your child have any psychiatric disorder?” and children with any psychiatric condition were not included in the study.

Written consent was obtained from parents.

### Study Size

To predict that the unhealthy nutrition rate in those with ADHD+ may have a minimum difference of 5% more than the unhealthy nutrition rate in those with ADHD; to be able to determine statistically significant with at most 5% Type I error margin and at least 80% power, it has been determined as a minimum sample size of 400. The determination of the sample size was calculated using the program “ www.e-picos.com,” NY, New York.

### Assessment

#### Food Consumption

A questionnaire containing processed meat products and ready-to-eat snacks were used. Among the foods consumed, attention was paid to whether they were homemade or not, and the foods that were bought ready-to-eat were questioned. The foods and groupings that are questioned while consuming food are listed below:

- Sujuk, salami, sausage, pastrami: Processed meat products
- Chocolate spread, sweets, jellybeans, chocolate, chewing gum: Chocolate-sweets
- Dessert, pastry, cake, biscuit: Bakery products
- Pudding, fruit yogurt, ice cream: Sugary and dairy products
- Chips, cracker: Salty snacks

Participants’ processed meat products and snack consumption, using a modified food frequency questionnaire form with 18 food, were questioned as “every day,” “once a week,” “2–3 days a week,” “3–4 days a week,” “5–6 days a week,” “2–3 days a month,” “once a month.” The frequency of food consumption was questioned along with the amount consumed, and the amount and frequency of these consumptions were determined. Foods with a consumption frequency of every day, 2–3 days a week, 3–4 days a week, and 5–6 days a week were labeled as “very often” and foods with a consumption frequency of once a week, 2–3 days a month, once a month, and the ones that have no consumption at all were labeled as “Very seldom or none.”

Food consumption amounts were taken by asking the various sizes of food sold in the market (small, medium, large), by showing photos, or by kitchen measurements (slices, spoons, bowls). Daily consumption amounts were calculated for each food according to the frequency of consumption. The amounts of foods were calculated in grams. The daily consumption of foods has not been evaluated on a product basis. Instead, the total amount of food consumption into classified food groups was calculated (amount of processed meat products consumption, amount of bakery products, etc.). By collecting all snacks, the total daily snack consumption amount was calculated.

#### ADHD Symptoms

##### Conners’ parent rating scale (CPRS) and Conners’ teacher rating scale (CTRS)

These scales are frequently used in behavior assessment. Based on the observations of the parents, the CPRS includes 48 questions used in the behavioral assessment of their children. Based on teachers “classroom observations, the CTRS consists of 28 questions used in students” behavioral evaluation [14].

##### Teacher and parent turgay DSM-IV-based child and adolescent disruptive behavioral disorders screening and rating scale (T-DSM-IV-S)

It is a scale developed according to DSM-IV criteria [15]. There are 9 questions about inattentiveness, 6 about hyperactivity, 3 about impulsivity, 8 about being oppositional, and 15 about behavioral. It is filled by parents and teachers of children with ADHD. For each point, there are 0: none, 1: a little, 2: more, 3: too many, options.

### Statistical Analysis

The Statistical Package for the Social Sciences version 17.0 was used for statistical evaluation of the data. To control the confoundings, hospitals and schools in the same neighborhood were preferred so that the income levels and living standards of the groups were similar to each other. To check that the questionnaire was conducted properly, questionnaires with meaningless or opposite answers were not evaluated. Similarly, sloppy questionnaires with the same answer to all questions were not taken into consideration. The arithmetic mean and standard deviation values of the data obtained from individuals were calculated. Nominal variables are given using frequency and percentages. Descriptive findings were presented as mean and standard deviation for continuous data. Independent sample t-Test was used for the average comparison between binary groups. Chi-square tests were used to compare the classified data. Chi square test was calculated using another program “ www.e-picos.com,” NY, New York. The relationships between the variables were examined with the Spearman correlation coefficient and binary regression analyses. The tests were determined in the 95% confidence interval, and the significance level was p<0.05.

## RESULTS

### Participants

Since the symptom scales of the case group were obtained from parents and teachers, 169 forms were distributed to them, 94 participants returned the CTRS form, 75 returned the CPRS form, 86 returned the teacher T-DSM-IV-S, and 73 participants returned the parent T-DSM-IV-S. As a result, demographic characteristics and food records were analyzed with 169 data from the case group and 221 from the control group. Besides, the relationships between symptom scales and food consumption in the case group were analyzed for children whose scales could be reached (CTRS: 94; CPRS: 75; Teacher T-DSM-IV-S: 86; Parent T-DSM-IV-S: 73).

### Demographic Features

The demographic features of the ADHD and control groups are shown in Table 1. The average age of all participants is 10±2 years, and there is no difference between ADHD and control groups. The percentage of girls in the control group is higher than the ADHD group (p<0.001). Moreover, the percentage of boys in the ADHD group is higher than the control group (p<0.001).

**Table 1:** Demographic features of ADHD and control group

Demographic features	ADHD (N:169)	Control (N:221)	Total (N:390)	p
Gender (%)				<0.001*
Girl	22.0	42.0	33.5	
Boy	78.0	58.0	66.4	
Age (years) (Mean±SD)	9.76±2.45	10.25±2.39	10.04±2.42	0.05
Age at diagnosis (%)				–
0–29 days	32.0	–	–	
1–5 months	27.0	–	–	
6 months and above	41.0	–	–	
Mother’s education (%)				
Primary education	52.4	68.9	61.8	<0.001*
High school	29.2	21.5	24.8	0.08
University	18.4	9.6	13.4	0.01*
Father’s education (%)				0.17
Primary education	49.7	56.1	53.8	
High school	34.3	33.9	34.4	
University	14.8	9.0	11.6	

ADHD: Attention-deficit and hyperactivity disorder; SD: Standard deviation; *: P<0.05, Chi-square test.

It was determined that most of the mothers (61.4%) had primary education, the rest had high school (24.8%) and university (13.4%) education, and the education level of fathers was slightly higher than mothers. The education level of the mothers of children with ADHD is higher than that of the control group (p<0.05).

The ADHD group is classified according to the time of diagnosis and treatment. The percentage of receiving treatment for ADHD patients is 32% for 0–29 days, 27% for 1–5 months, and 41% for 6 months and above.

### Food Consumption

The frequency of food consumption was evaluated between the two groups (Table 2). Pastrami consumption was not included in the analysis as it was not at a statistically significant level. For this reason, 17 of the 18 foods were analyzed. The frequency of sujuk and cake consumption among patients with ADHD is higher than controls (p<0.05).

**Table 2:** Frequency of food intakes between children with ADHD and controls

Food	ADHD		Control		p
	Very seldom or none (%)	Very often (%)	Very seldom or none (%)	Very often (%)	
Sujuk	58.6	41.4	80.0	20.0	0.000*
Salami	76.3	23.7	83.0	16.3	0.68
Sausage	95.9	4.1	95.0	5.0	0.88
Chocolate spread	52.1	47.9	48.0	52.0	0.41
Sweets	82.2	17.8	82.4	17.6	0.97
Jellybeans	89.3	10.7	93.2	6.8	0.24
Dessert	93.5	6.5	93.2	6.8	1.00
Pastry	96.4	3.6	95.5	5.0	0.82
Cake	73.4	26.6	84.6	15.4	0.006*
Pudding	85.2	14.8	84.6	15.4	0.82
Fruit yogurt	92.9	7.1	91.4	8.6	0.72
Chocolate	32.5	67.5	38.9	61.1	0.19
Biscuit	68.0	32.0	64.7	35.3	0.49
Chewing gum	58.6	41.4	55.2	44.8	0.50
Ice cream	65.7	34.3	58.4	41.6	0.14
Chips	69.2	30.8	75.6	24.4	0.16
Cracker	70.4	29.6	76.0	24.0	0.21

ADHD: Attention-deficit and hyperactivity disorder; *: P<0.05, Chi-square test.

The amount of processed meat products, bakery products, sugary and dairy products, chocolate-sweets, salty snacks, and all snacks consumption was compared (Table 3). The average amount of processed meat products, sugary and dairy products, chocolate-sweets consumption in the ADHD group is higher than the control group (p<0.05).

**Table 3:** Daily consumption amount of food intake between children with ADHD and controls

Food	ADHD Mean (SD)	Control Mean (SD)	p
Processed meat products (g)	27.22 (38.21)	15.32 (20.28)	0.000*
Bakery products (g)	37.86 (46.49)	41.15 (54.59)	0.53
Sugary and dairy products (g)	57.78 (85.50)	41.63 (60.69)	0.03*
Chocolate-sweets (g)	61.11 (65.42)	37.09 (34.61)	0.000*
Salty snacks (g)	24.60 (40.11)	20.28 (28.40)	0.21
All snacks (g)	236.77 (218.82)	260.77 (183.82)	0.24

ADHD: Attention-deficit and hyperactivity disorder; SD: Standard deviation; *: P<0.05, Independent Sample t-Test.

Binary regression was analyzed to determine the effect of age, gender, and parents’ education on food consumption (Tables 4–7). The ages were grouped as 6–10 and 11–17 years, and there was no relationship between age groups and food consumption. Parents’ education was categorized as primary education = low education; high school and university = high education, and there was no relationship between parents’ education and food consumption. Similarly, the gender of children with ADHD did not affect their food consumption.

**Table 4:** Binary regression analyses for the age factors associated with food consumption in children with ADHD

Food	p	OR	95.0% CI	
			Lower	Upper
Sujuk (f)	0.05	0.476	0.224	1.015
Cake (f)	0.77	0.896	0.427	1.881
Processed meat products (a)	0.87	1.001	0.990	1.012
Sugary and dairy products (a)	0.75	1.001	0.996	1.005
Chocolate-sweets (a)	0.82	1.001	0.995	1.006

ADHD: Attention-deficit and hyperactivity disorder; OR: Odds ratio; CI: Confidence interval; a: Consumption amount; f: Consumption frequency.

**Table 5:** Binary regression analyses for the gender factors associated with food consumption in children with ADHD

Food	p	OR	95.0% CI	
			Lower	Upper
Sujuk (f)	0.55	1.339	0.511	3.508
Cake (f)	0.25	0.579	0.227	1.474
Processed meat products (a)	0.06	0.980	0.960	1.001
Sugary and dairy products (a)	0.75	1.001	0.996	1.006
Chocolate-sweets (a)	0.36	1.003	0.997	1.009

ADHD: Attention-deficit and hyperactivity disorder; OR: Odds ratio; CI: Confidence interval; a: Consumption amount; f: Consumption frequency.

**Table 6:** Binary regression analyses for the mother’s education factors associated with food consumption in children with ADHD

Food	p	OR	95.0% CI	
			Lower	Upper
Sujuk (f)	0.16	0.907	0.791	1.040
Cake (f)	0.10	1.107	0.979	1.252
Processed meat products (a)	0.41	1.004	0.994	1.015
Sugary and dairy products (a)	0.27	0.998	0.993	1.002
Chocolate-sweets (a)	0.12	0.996	0.990	1.001

ADHD: Attention-deficit and hyperactivity disorder; OR: Odds ratio; CI: Confidence interval; a: Consumption amount; f: Consumption frequency.

**Table 7:** Binary regression analyses for the father’s education factors associated with food consumption in children with ADHD

Food	p	OR	95.0% CI	
			Lower	Upper
Sujuk (f)	0.85	0.907	0.791	1.040
Cake (f)	0.10	1.107	0.979	1.252
Processed meat products (a)	0.41	1.004	0.994	1.015
Sugary and dairy products (a)	0.27	0.998	0.993	1.002
Chocolate-sweets (a)	0.12	0.996	0.990	1.001

ADHD: Attention-deficit and hyperactivity disorder; OR: Odds ratio; CI: Confidence interval; a: Consumption amount; f: Consumption frequency.

### Teacher and Parent T-DSM-IV-S, CPRS, and CTRS

The relationships between total scores and food intakes were examined with the Spearman correlation test in children with ADHD (Table 8). A positive correlation was found between the consumption amount of all snacks, amount of chocolate-sweets and frequency of sujuk, chocolate, sweets, jellybeans, dessert, pastry, and chocolate spread, and ADHD total scores (p<0.05).

**Table 8:** Correlation between the four total scores and food intakes children with ADHD

Food	CTRS		CPRS		Teacher T-DSM-IV		Parent T-DSM-IV-S	
	r	p	r	p	r	p	r	p
All snacks (a)	0.149	0.15	0.280	0.01*	0.096	0.37	0.176	0.13
Chocolate-sweets (a)	0.038	0.71	0.367	0.001*	0.137	0.20	0.229	0.05
Sujuk (f)	0.229	0.02*	-0.43	0.08	0.122	0.26	-0.021	0.86
Chocolate (f)	0.206	0.04*	0.047	0.68	0.119	0.27	-0.009	0.94
Sweets (f)	0.168	0.10	0.231	0.04*	0.248	0.02*	0.229	0.05
Jellybeans (f)	-0.74	0.48	0.252	0.02*	-0.046	0.67	0.125	0.29
Dessert (f)	-0.134	0.19	0.261	0.02*	-0.098	0.36	0.238	0.04*
Pastry (f)	-0.024	0.81	0.250	0.02*	0.078	0.47	0.229	0.05
Chocolate spread (f)	0.197	0.05	0.181	0.12	0.219	0.04*	0.174	0.14

ADHD: Attention-deficit and hyperactivity disorder; CTRS: Conners’ teacher rating scale; CPRS: Conners’ parent rating scale; *: P<0.05, r: Spearman correlation; a: Consumption amount, f: Consumption frequency.

## DISCUSSION

Since ADHD is the most common neurodevelopmental disorder of childhood [1], studies related to its prevention and treatment are the subject of current research. In this study, it has been found that children with ADHD have a higher consumption of processed meat products and snacks compared to healthy controls. Moreover, this type of food consumption has been found to be associated with ADHD symptoms.

Western-style diet, where processed meats are consumed more, has been associated with ADHD [2]. In our study, the consumption of processed meat products was compared between ADHD and the control group. It has been found that children with ADHD consume statistically significantly more frequent sujuks and more processed meat products. In a study similar to our study, it was shown that the western-style diet score, which includes processed meat products, was higher in the ADHD group compared to the control [13]. Liu et al. [14] reported a positive correlation between the consumption of processed meat products and hyperactivity score.

Instant food, which is described as “junk food,” is snacks that are high in energy density and poor in terms of nutrients [15]. It can be considered unhealthy because it contains a lot of energy, saturated fat, and sugar. In our study, it was found that the frequency of consumption of cake and the amount of sugary and dairy products and chocolate-sweets was higher in the group with ADHD. Similarly, Wang et al. [16], reported that the group with ADHD consumes food rich in sugar and fat more. In another study, it was observed that ADHD consumed ready-to-eat cakes, sweets, and candies more than the control group [17]. In our study, there was a positive correlation between ADHD symptoms and sujuk and all snacks. In line with the findings of our study, in a China study, there was a positive correlation between processed and snack labeled food and ADHD symptoms [18]. Wolff et al. [19] found that children with ADHD consume more candies and sugary gum and that their hyperactivity scores are related to these foods.

Various hypotheses regarding the unhealthy diet of patients with ADHD have been proposed. First of all, it is the impulsivity and inattention components of ADHD that can be effective in an unhealthy diet. Easy and cheap access to fatty and sugary food in the markets is one of the important factors affecting the selection of these foods. People with high levels of impulsivity often have difficulty in resisting conditions that are offered to them and, are more sensitive to rewards [23]. Guerrieri et al. [20] observed that more caloric intake occurred in children with high impulsivity against the food of varying color, shape, taste, and texture. It is stated that children with ADHD tend to reach foods that are easily accessible and practical to eat. Also, they were shown to choose the fastest option between the fast-food option and the delayed food option [21]. Inattention and lack of planning can cause difficulties in regular diet and adherence to diet regimens. In addition, inattentiveness can be associated with a lack of awareness of food intake. Besides, abnormal eating patterns may also be included in disturbing behaviors related to hyperactivity [22].

Parents’ attitudes should be taken into consideration when interpreting children’s unhealthy food liabilities in ADHD. Studies report strong correlations between parental behavior and children’s food consumption [23]. Genetic factors are known to be effective in ADHD, and it is estimated that at least one child of half of the adults with ADHD has ADHD. Inattention and difficulties in self-control in the parent of ADHD can lead to neglect against the needs of the child [24]. Since families with ADHD symptoms can show more negligent parental behaviors, these behaviors can lead to the child’s consumption of food in a comfortable and unrestricted manner. Besides, due to the impulsive choices of families with ADHD, the possibility of having unhealthy food at home may increase. Easy access to unhealthy food has been associated with an increase in snack consumption in children [25].

Parenting a child with ADHD is a tiring, time-consuming, and emotional process that involves feelings of guilt and hopelessness at different stages of life. The daily life of a parent with ADHD is full of plans to overcome daily challenges with his child and be prepared for these challenges [26]. Studies with parents of children with ADHD have been shown to have higher stress levels [27–29]. It is time and patience for a tired parent to educate and guide her/his ADHD child on healthy eating. For this reason, children with ADHD may be at risk for unhealthy nutrition.

Our results can partially be generalized. Because foods are produced generally in similar ways. Ingredients in processed foods are nearly the same. ADHD symptoms are also the same all over the world. Hence, it is expected that the relation of consumption of processed foods and ADHD can be similar also in other populations.

### Limitations of the Research

- The parents in the control group were unable to participate in oral interviews. The parents’ written information was considered.
- Failure to take the scales used to evaluate ADHD symptoms from all participants.
- In both groups, it was not questioned whether the parents had a diagnosis of ADHD. This situation can be a potential confounding factor because of food choice, accessibility and consumption. Like this factor, some other variables that can cause ADHD were not evaluated in the study.

## Conclusion

In our research, processed meat products and snack consumption were found to be higher than healthy peers. Foods were found to be associated with the total ADHD score.

This study shows that children with ADHD are at risk for unhealthy nutrition. Children with ADHD should be under more observation in terms of nutrition than healthy children. It should be particularly recommended that both healthy children and children with ADHD reduce the consumption of this type of unhealthy food. Research is needed to show a clear mechanism for the causes of these results.

## References

[1] Martin A, Bloch MR, Volkmar FR (2018). Attention-deficit hyperactivity disorder. Lewis’s Child and Adolescent Psychiatry. A Comprehensive Textbook.

[2] Howard AL, Robinson M, Smith GJ, Ambrosini GL, Piek JP, Oddy WH (2011). ADHD is associated with a “Western” dietary pattern in adolescents. J Atten Disord.

[3] Marwitz SE, Woodie LN, Blythe SN (2015). Western-style diet induces insulin insensitivity and hyperactivity in adolescent male rats. Physiol Behav.

[4] Greenwood CE, Winocur G (2001). Glucose treatment reduces memory deficits in young adult rats fed high-fat diets. Neurobiol Learn Mem.

[5] Beilharz JE, Maniam J, Morris MJ (2014). Short exposure to a diet rich in both fat and sugar or sugar alone impairs place, but not object recognition memory in rats. Brain Behav Immun.

[6] Kanoski SE, Davidson TL (2011). Western diet consumption and cognitive impairment: links to hippocampal dysfunction and obesity. Physiol Behav.

[7] Akbaraly TN, Singh-Manoux A, Marmot MG, Brunner EJ (2009). Education attenuates the association between dietary patterns and cognition. Dement Geriatr Cogn Disord.

[8] Holton KF, Johnstone JM, Brandley ET, Nigg JT (2019). Evaluation of dietary intake in children and college students with and without attention-deficit/hyperactivity disorder. Nutr Neurosci.

[9] Holloway CJ, Cochlin LE, Emmanuel Y, Murray A, Codreanu I, Edwards LM (2011). A high-fat diet impairs cardiac high-energy phosphate metabolism and cognitive function in healthy human subjects. Am J Clin Nutr.

[10] Yeomans MR (2017). Adverse effects of consuming high fat-sugar diets on cognition: implications for understanding obesity. Proc Nutr Soc.

[11] Şener Ş, Dereboy Ç, Dereboy İF, Sertcan Y (1995). Conners Öğretmen Derecelendirme Ölçeği Türkçe uyarlaması. Çocuk ve Gençlik Ruh Sağlığı Derg.

[12] Turgay A (1994). Disruptive behavior disorders:child and adolescent screening and ratingscales for children, adolescents, parents andteachers.

[13] Abbasi K, Beigrezai S, Ghiasvand R, Pourmasoumi M, Mahaki B (2019). Dietary patterns and attention deficit hyperactivity disorder among Iranian children: a case-control study. J Am Coll Nutr.

[14] Liu J, He P, Li L, Shen T, Wu M, Hu J (2014). Study on the association between diet, nutrient and attention deficit hyperactivity disorder among children in Shanghai, Kunshan, Wuxi three kindergarten. [Article in Chinese]. Wei Sheng Yan Jiu.

[15] Larson N, Miller JM, Eisenberg ME, Watts AW, Story M, Neumark-Sztainer D (2017). Multicontextual correlates of energy-dense, nutrient-poor snack food consumption by adolescents. Appetite.

[16] Wang LJ, Yu YH, Fu ML, Yeh WT, Hsu JL, Yang YH (2019). Dietary profiles, nutritional biochemistry status, and attention-deficit/hyperactivity disorder: path analysis for a case-control study. J Clin Med.

[17] San Mauro Martín I, Blumenfeld Olivares JA, Garicano Vilar E, Echeverry López M, García Bernat M, Quevedo Santos Y (2018). Nutritional and environmental factors in attention-deficit hyperactivity disorder (ADHD): A cross-sectional study. Nutr Neurosci.

[18] Yan S, Cao H, Gu C, Ni L, Tao H, Shao T (2018). Dietary patterns are associated with attention-deficit/hyperactivity disorder (ADHD) symptoms among preschoolers in mainland China. Eur J Clin Nutr.

[19] Wolff N, Reimelt C, Ehrlich S, Hölling H, Mogwitz S, Roessner V (2019). On the positive association between candy and fruit gum consumption and hyperactivity in children and adolescents with ADHD. Z Kinder Jugendpsychiatr Psychother.

[20] Guerrieri R, Nederkoorn C, Jansen A (2008). The interaction between impulsivity and a varied food environment: its influence on food intake and overweight. Int J Obes (Lond).

[21] Phillips W (2014). Nutrition management of children with attention deficit hyperactivity disorder. Infant, Child, Adolesc Nutr.

[22] Cortese S, Moreira-Maia CR, St Fleur D, Morcillo-Peñalver C, Rohde LA, Faraone SV (2016). Association between ADHD and obesity: a systematic review and meta-analysis. Am J Psychiatry.

[23] Yee AZ, Lwin MO, Ho SS (2017). The influence of parental practices on child promotive and preventive food consumption behaviors: a systematic review and meta-analysis. Int J Behav Nutr Phys Act.

[24] Park JL, Hudec KL, Johnston C (2017). Parental ADHD symptoms and parenting behaviors: A meta-analytic review. Clin Psychol Rev.

[25] Blaine RE, Kachurak A, Davison KK, Klabunde R, Fisher JO (2017). Food parenting and child snacking: a systematic review. Int J Behav Nutr Phys Act.

[26] Laugesen B, Groenkjaer M (2015). Parenting experiences of living with a child with attention deficit hyperactivity disorder: a systematic review of qualitative evidence. JBI Database System Rev Implement Rep.

[27] Pimentel MJ, Vieira-Santos S, Santos V, Vale MC (2011). Mothers of children with attention deficit/hyperactivity disorder: relationship among parenting stress, parental practices and child behaviour. Atten Defic Hyperact Disord.

[28] Graziano PA, McNamara JP, Geffken GR, Reid A (2011). Severity of children’s ADHD symptoms and parenting stress: a multiple mediation model of self-regulation. J Abnorm Child Psychol.

[29] Hernández-Otero I, Doddamani L, Dutray B, Gagliano A, Haertling F, Bloomfield R (2015). Stress levels experienced by parents of children with and without attention-deficit/hyperactivity disorder during the back-to-school period: results of a European and Canadian survey. Int J Psychiatry Clin Pract.

